# The Efficacy and Safety of Sacubitril/Valsartan Compared to Valsartan in Patients with Heart Failure and Mildly Reduced and Preserved Ejection Fractions: A Systematic Review and Meta-Analysis of Randomized Controlled Trials

**DOI:** 10.3390/jcm13061572

**Published:** 2024-03-09

**Authors:** Sharath Kommu, Richard L. Berg

**Affiliations:** 1 Department of Hospital Medicine, Marshfield Clinic Health System, Rice Lake, WI 54868, USA; 2 Department of Medicine, UW School of Public Health and Medicine, Madison, WI 53705, USA; 3 Office of Research Computing and Analytics, Marshfield Clinic Research Institute, Marshfield, WI 54449, USA

**Keywords:** sacubitril/valsartan, sacubitril/valsartan, angiotensin receptor-neprilysin inhibitor, ARNI, valsartan, heart failure with preserved ejection fraction, HFpEF, heart failure with mildly reduced ejection fraction, HFmrEF, heart failure

## Abstract

**Background**: Sacubitril/valsartan improves heart failure (HF) outcomes in patients with heart failure with reduced ejection fraction (HFrEF). However, randomized controlled trials (RCTs) in patients with heart failure and mildly reduced ejection fraction (HFmrEF) and heart failure with preserved ejection fraction (HFpEF) have shown inconsistent results. We conducted this meta-analysis to comprehensively evaluate the efficacy and safety of sacubitril/valsartan compared to valsartan within this specific patient population. **Methods**: We searched the MEDLINE database and ClinicalTrials.gov and identified four RCTs that could be included in our analysis, with 3375 patients in the sacubitril/valsartan group and 3362 in the valsartan group. **Results**: Our study shows that, in patients with HFmrEF and HFpEF, sacubitril/valsartan was superior to valsartan in some of the key HF outcomes, such as the Kansas City Cardiomyopathy Questionnaire Clinical Summary Score (KCCQ CSS), with a small but significant mean difference of 1.13 (95% confidence interval or CI of 0.15 to 2.11, *p*-value 0.024), an improvement in the New York Heart Association (NYHA) class (odds ratio or OR of 1.32, 95% CI 1.11 to 1.58, *p*-value 0.002), and the composite outcome of hospitalizations for HF and cardiovascular death, with a relative risk (RR) of 0.86 (95% CI 0.75 to 0.99, *p*-value 0.04). However, there was no additional benefit with sacubitril/valsartan compared to valsartan for the outcomes of cardiovascular death and all-cause mortality. In terms of side effects, sacubitril/valsartan was associated with a higher risk of hypotension when compared to valsartan (OR 1.67, 95% CI 1.27 to 2.19, *p*-value < 0.0001), but did not show an increased risk of hyperkalemia or worsening renal function. **Conclusions**: In individuals with HFmrEF or HFpEF, sacubitril/valsartan can result in improvements in the HF outcomes of the KCCQ CSS, the NYHA class, and the composite outcome of hospitalization for HF and cardiovascular death when compared to valsartan. While there was a higher risk of hypotension with sacubitril/valsartan compared to valsartan, there was no corresponding increase in the risk of hyperkalemia or worsening renal function.

## 1. Introduction

Cardiovascular diseases impose a substantial healthcare burden and stand as the leading cause of mortality worldwide [1]. Within this category, heart failure (HF) stands out as a significant contributor to morbidity and mortality, affecting over 64 million individuals globally [2]. In high-income nations, the prevalence of HF varies from 1% to 3% among the general adult population [2]. Improved diagnostic technologies and treatment modalities are anticipated to increase this prevalence [2].

Researchers have recognized neprilysin as having hydrolyzing activity on atrial natriuretic peptide (ANP), bradykinin, adrenomedullin, and various vasoactive peptides [3,4,5,6]. The breakdown of ANP impedes its potent vasodilatory, diuretic, and natriuretic effects. Consequently, inhibiting neprilysin would reverse these effects, positively impacting HF. Sacubitril serves as a neprilysin inhibitor, and combining it with an angiotensin receptor blocker (ARB) like valsartan could yield additional benefits. Clinical trials exploring the effects of the angiotensin receptor-neprilysin inhibitor (ARNI), which is sacubitril/valsartan in patients with heart failure with reduced ejection fraction (HFrEF) with an ejection fraction (EF) of ≤40% have demonstrated positive outcomes in enhancing HF management [6,7,8]. Recognizing these benefits, the American Heart Association (AHA), American College of Cardiology (ACC), and Heart Failure Society of America (HFSA) guidelines have integrated ARNI into guideline-directed medical therapy (GDMT) for managing HFrEF [9].

In light of the positive outcomes observed with sacubitril/valsartan therapy in patients with HFrEF, researchers have conducted randomized controlled trials (RCTs) investigating the impact of sacubitril/valsartan in patients with heart failure with mid-range or mildly reduced ejection fraction (HFmrEF) with an EF from 41 to 49% and heart failure with preserved ejection fraction (HFpEF) with an EF ≥ 50% [10,11,12,13]. However, these RCTs have yielded conflicting results regarding the efficacy of sacubitril/valsartan in these specific patient populations, and concerns about medication side effects have emerged. A meta-analysis pooling the existing randomized controlled trials involving patients with HFmrEF and HFpEF holds promise for a comprehensive analysis. Such an approach may answer questions regarding the efficacy and suitability of sacubitril/valsartan in this patient cohort.

While a few meta-analyses have attempted to study the effect of sacubitril/valsartan in patients with HFmrEF and HFpEF, these analyses have often included the HFrEF population, making it challenging to draw distinct conclusions for HFmrEF and HFpEF cohorts. To the best of our knowledge, there is a notable absence of meta-analyses focused exclusively on assessing the efficacy and safety of sacubitril/valsartan in patients with HFmrEF and HFpEF. To address this knowledge gap, we undertook the present study. Our research offers valuable insights into sacubitril/valsartan’s potential advantages and safety profile in these specific HF cohorts of HFmrEF and HFpEF.

## 2. Materials and Methods

### 2.1. Search Strategy and Study Selection

For individuals with chronic symptomatic HFrEF treated with angiotensin-converting enzyme inhibitors (ACEi) or ARBs, the guidelines suggest transitioning to ARNI therapy [9]. Similarly, researchers have compared ACEi and/or ARBs with ARNI therapy in patients with HFpEF and HFmrEFs. Within the ACEi and ARB classes, ARBs are noted for having a more favorable side effect profile and being better tolerated. Studies investigating ARNIs versus ARBs often focus on valsartan as the representative ARB. Consequently, we opted to examine trials comparing sacubitril/valsartan with valsartan specifically.

We used the PRISMA (Preferred Reporting Items for Systematic Reviews and Meta-Analyses) guidelines to identify the studies for this meta-analysis. This study is not registered under any platform.

We searched the online databases MEDLINE (using PubMed) and ClinicalTrials.gov between January 2012 and November 2023 using the search terms: angiotensin receptor-neprilysin inhibitor, ARNI, sacubitril/valsartan, heart failure, and heart failure with preserved ejection fraction. Studies were considered to be eligible if they fulfilled all the following criteria: (1) they adopted a randomized controlled design involving human participants and were published in English; (2) the study population comprised patients diagnosed with HFpEF or HFmrEF; (3) they examined the effects of sacubitril/valsartan and valsartan; and (4) they analyzed outcomes related to heart failure. Any studies failing to meet these criteria were excluded from the analysis. Online search results were reviewed for potential inclusion in the meta-analysis. The references of the identified studies were manually reviewed to identify any potential additional studies.

### 2.2. Data Extraction

Upon the identification of eligible studies for inclusion, pertinent data for our meta-analysis were extracted. These included details such as the study name, publication year, study population characteristics, study duration, ejection fraction, the number of patients in the sacubitril/valsartan and valsartan groups, HF outcomes, side effects, and the frequency of events associated with outcomes and side effects. 

### 2.3. Quality Assessment

To assess the quality of the RCTs, the Cochrane risk-of-bias tool for randomized trials, revised version 2 (RoB2), was employed [14]. The risk of bias was categorized as low risk, some concerns, or high risk.

### 2.4. Statistical Analysis

The data were analyzed using the meta package in R software (version 4.3.1) with the inverse variance method [15]. Two measures of heterogeneity were calculated to evaluate the need for random effects: Higgins and Thompson’s I^2^ statistic and the heterogeneity variance τ^2^. If the *p*-value for heterogeneity was <0.1, a random effects model was used. A calculated *p*-value of <0.05 was considered to be significant evidence of a treatment difference.

## 3. Results

**Figure 1:**
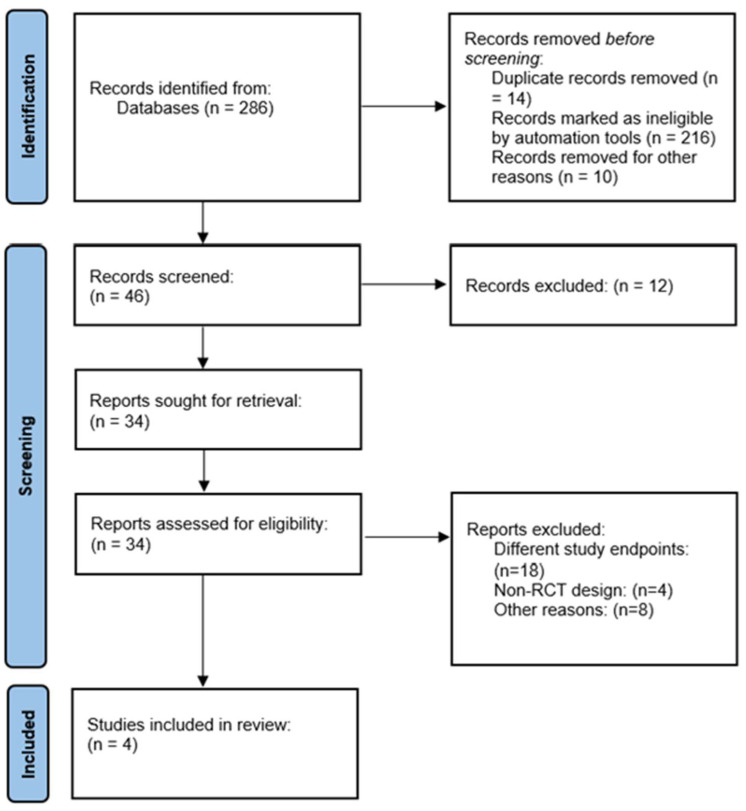
PRISMA algorithm, showing the selection of studies for this meta-analysis. PRISMA—Preferred Reporting Items for Systematic Reviews and Meta-Analyses.

**Figure 2:**
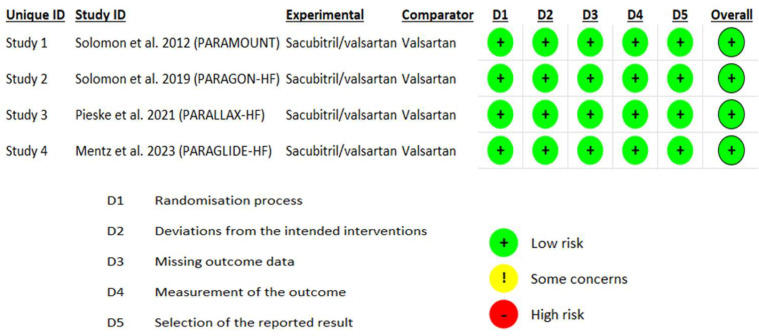
The risk of bias for the four studies, assessed using version 2 of the Cochrane risk-of-bias tool (ROB2) [10,11,12,13].

The online data search resulted in 286 articles. Using the PRISMA algorithm and incorporating our inclusion criteria, four studies that could be included in the meta-analysis were identified (Figure 1). According to the revised Cochrane risk-of-bias tool for randomized trials (RoB2), no major risk of bias was found in any of the four studies, indicating the overall reliability of the studies selected. (Figure 2). 

The four studies included in this meta-analysis are the PARAMOUNT trial by Solomon et al. [10], Prospective Comparison of ARNI with ARB Global Outcomes in HF with Preserved Ejection Fraction (PARAGON-HF) trial by Solomon et al. [11], Prospective Comparison of ARNI vs. Comorbidity-Associated Conventional Therapy on Quality of Life and Exercise Capacity (PARALLAX) trial by Pieske et al. [12], and the Prospective comparison of ARNI with ARB Given following stabiLization In DEcompensated HFpEF (PARAGLIDE-HF trial) by Mentz et al. [13]. From these four studies, a total of 6737 patients are included in this meta-analysis, with 3375 patients in the sacubitril/valsartan group and 3362 in the valsartan group. Information about the characteristics of these studies is included in Table 1.

The shared outcomes and significant side effects across the four studies were analyzed. The HF parameters studied included the Kansas City Cardiomyopathy Questionnaire Clinical Summary Score (KCCQ CSS), improvements in the New York Heart Association (NYHA) class, and the composite of hospitalizations for HF and cardiovascular death, as well as the specific outcomes of hospitalizations for HF and cardiovascular death. The major side effects common to these studies included hypotension, hyperkalemia, and worsening renal function. The analysis of these outcomes and side effects is detailed below.

**Figure 3:**
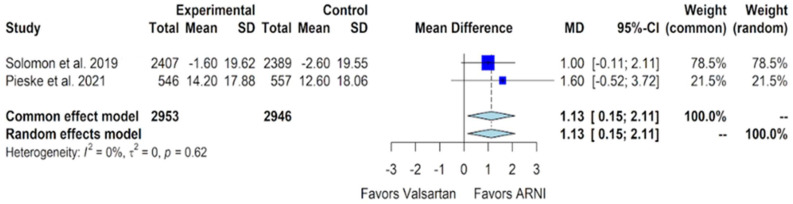
Forest plot showing the change in KCCQ CSS with sacubitril/valsartan compared to valsartan in patients with HFmrEF and HFpEF. KCCQ CSS—Kansas City cardiomyopathy questionnaire clinical summary score, HFmrEF—heart failure with mildly reduced ejection fraction, HFpEF—heart failure with preserved ejection fraction, and ARNI—angiotensin receptor-neprilysin inhibitor, SD—standard deviation, MD—mean difference, CI—confidence interval. In the forest plot, dark blue squares represent the point estimates, and the size of the square is a function of the weight given to each study in the meta-analysis. Horizontal solid black lines represent 95% CI. The bottom light blue diamonds represent the summary estimates, with the width of the diamond illustrating the 95% CI [11,12].

The KCCQ CSS is a patient-reported outcome score (on a 0–100 scale) that measures the symptoms and physical limitations associated with heart failure, with higher scores indicating better symptoms and physical functioning. For the outcome of improvement in the KCCQ CSS, among the eligible studies, only two provided pertinent data for inclusion in our meta-analysis. In the PARAGON-HF trial, an eight-month treatment period revealed a mean decrease in the KCCQ CSS of 1.6 points in the sacubitril/valsartan group, compared to a mean decrease of 2.6 points in the valsartan group. Although both groups experienced a reduction in KCCQ CSS, the sacubitril/valsartan group demonstrated a more favorable mean change by 1.0 points (95% CI of 0.0 to 2.1) [11]. Conversely, the PARALLAX trial showed an improved KCCQ CSS at 24 weeks for both the sacubitril/valsartan and valsartan groups. The adjusted mean change from baseline in the sacubitril/valsartan group was 14.2; in the valsartan group, it was 12.6. The adjusted mean difference was 1.6 (95% CI of 0.6 to 3.7) [12]. Upon combining the results of both studies through a pooled analysis, a favorable KCCQ CSS was observed for sacubitril/valsartan compared to valsartan, with a mean difference of 1.13 (95% CI of 0.15 to 2.11, *p*-value of 0.024) (Figure 3).

**Figure 4:**
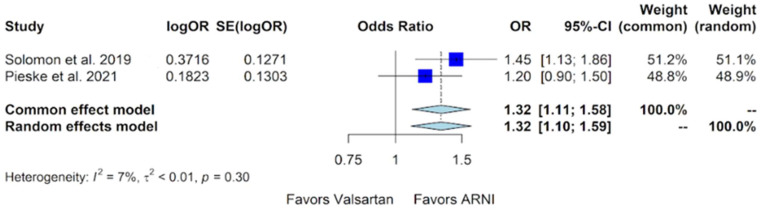
Forest plot showing the participants with improved NYHA class with sacubitril/valsartan compared to valsartan in patients with HFmrEF and HFpEF. NYHA—New York Heart Association, HFmrEF—heart failure with mildly reduced ejection fraction, HFpEF—heart failure with preserved ejection fraction, and ARNI—angiotensin receptor-neprilysin inhibitor, OR—odds ratio, SE—standard error, CI—confidence interval. In the forest plot, dark blue squares represent the point estimates, and the size of the square is a function of the weight given to each study in the meta-analysis. Horizontal solid black lines represent 95% CI. The bottom light blue diamonds represent the summary estimates, with the width of the diamond illustrating the 95% CI [11,12].

The assessment of the number of participants experiencing an improved NYHA class was feasible from two trials. In the PARAGON-HF trial, 347 out of 2316 patients (15%) in the sacubitril/valsartan group demonstrated an NYHA class improvement, compared to 289 out of 2302 patients (12.6%) in the valsartan group, at 8 months [11]. In the PARALLAX trial, the data at 24 weeks indicated that 146 out of 557 patients (26.2%) in the sacubitril/valsartan group showed an improvement in the NYHA class, compared to 138 out of 567 patients (24.3%) in the valsartan group [12]. Combining the data from both studies showed a significant improvement in NYHA class in the sacubitril/valsartan group compared to valsartan, with an odds ratio (OR) of 1.32 (95% CI of 1.10 to 1.59, *p*-value of 0.002) (Figure 4).

**Figure 5:**
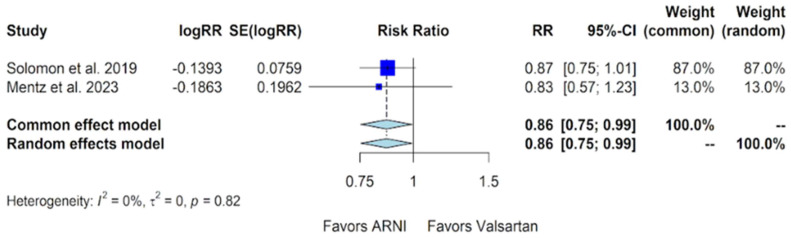
Forest plot showing the composite outcome of hospitalizations for heart failure and cardiovascular death with sacubitril/valsartan compared to valsartan in patients with HFmrEF and HFpEF. HFmrEF—heart failure with mildly reduced ejection fraction, HFpEF—heart failure with preserved ejection fraction, and ARNI—angiotensin receptor-neprilysin inhibitor, RR—relative risk, SE—standard error, CI—confidence interval. In the forest plot, dark blue squares represent the point estimates, and the size of the square is a function of the weight given to each study in the meta-analysis. Horizontal solid black lines represent 95% CI. The bottom light blue diamonds represent the summary estimates, with the width of the diamond illustrating the 95% CI [11,13].

The data concerning the composite outcomes of hospitalization for HF and cardiovascular death from two studies incorporated in this meta-analysis were considered. In the PARAGON-HF trial, there were 894 events out of 2407 patients with a rate of 12.8/100 patient years in the sacubitril/valsartan group, compared to 1009 events out of 2389 patients with a rate of 14.6/100 patient years in the valsartan group [11]. The PARAGLIDE-HF trial assessed the composite of HF hospitalizations, cardiovascular deaths, and urgent HF visits. In this trial, there were 94 such events out of 233 patients with a rate of 63.5/100 patient years in the sacubitril/valsartan group, compared to 117 events out of 233 patients with a rate of 76.2/100 patient years in the valsartan group [13]. Upon combining the data from both studies, this meta-analysis indicates a significant benefit with sacubitril/valsartan compared to valsartan in the composite outcome of hospitalizations for HF and cardiovascular death, with a relative risk (RR) of 0.86 (95% CI of 0.75 to 0.99, *p*-value of 0.04) (Figure 5).

**Figure 6:**
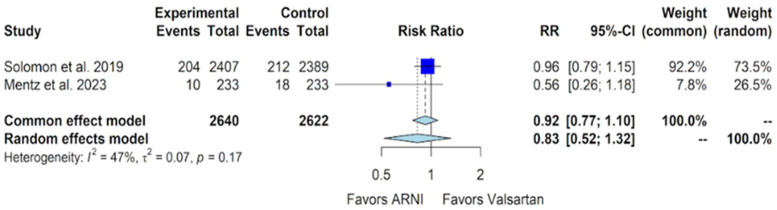
Forest plot showing the outcome of cardiovascular death with sacubitril/valsartan compared to valsartan in patients with HFmrEF and HFpEF. HFmrEF—heart failure with mildly reduced ejection fraction, HFpEF—heart failure with preserved ejection fraction, and ARNI—angiotensin receptor-neprilysin inhibitor, RR – relative risk, CI – confidence interval. In the forest plot, dark blue squares represent the point estimates, and the size of the square is a function of the weight given to each study in the meta-analysis. Horizontal solid black lines represent 95% CI. The bottom light blue diamonds represent the summary estimates, with the width of the diamond illustrating the 95% CI [11,13].

The outcome of cardiovascular death, another parameter included in this meta-analysis, had data from two trials. In the PARAGON-HF trial, there were 204 cardiovascular deaths out of 2407 patients in the sacubitril/valsartan group compared to 212 cardiovascular deaths out of 2389 patients in the valsartan group [11]. In contrast, the PARAGLIDE-HF trial reported 10 cardiovascular deaths out of 233 patients in the sacubitril/valsartan group, compared to 18 cardiovascular deaths out of 233 patients in the valsartan group [13]. Upon pooling the analysis of this data, no significant benefit in terms of cardiovascular death was observed in the sacubitril/valsartan group compared to the valsartan group, with a relative risk (RR) of 0.92 (95% CI of 0.77 to 1.10, *p*-value of 0.38) (Figure 6).

**Figure 7:**
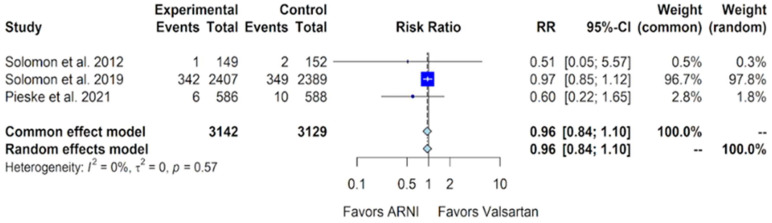
Forest plot showing the outcome of all-cause mortality with sacubitril/valsartan compared to valsartan in patients with HFmrEF and HFpEF. HFmrEF—heart failure with mildly reduced ejection fraction, HFpEF—heart failure with preserved ejection fraction, and ARNI—angiotensin receptor-neprilysin inhibitor. RR—relative risk, CI—confidence interval. In the forest plot, dark blue squares represent the point estimates, and the size of the square is a function of the weight given to each study in the meta-analysis. Horizontal solid black lines represent 95% confidence intervals (CI). The bottom light blue diamonds represent the summary estimates, with the width of the diamond illustrating the 95% CI [10,11,12].

The outcome of all-cause mortality, reported in three of the four trials, is also evaluated here. In the PARAMOUNT trial, 1 death occurred out of 149 patients in the sacubitril/valsartan group compared to 2 deaths out of 152 patients in the valsartan group [10]. The PARAGON-HF trial reported 342 deaths out of 2407 patients (14.2%) in the sacubitril/valsartan group, compared to 349 deaths out of 2407 patients (14.6%) in the valsartan group [11]. In the PARALLAX trial, there were 6 deaths out of 586 patients (1%) in the sacubitril/valsartan group, compared to 10 out of 588 (1.7%) in the valsartan group [12]. A pooled analysis of these data reveals no additional benefit in terms of all-cause mortality with sacubitril/valsartan compared to valsartan, showing a relative risk (RR) of 0.96 (95% CI of 0.85 to 1.10, *p*-value of 0.56) (Figure 7).

**Figure 8:**
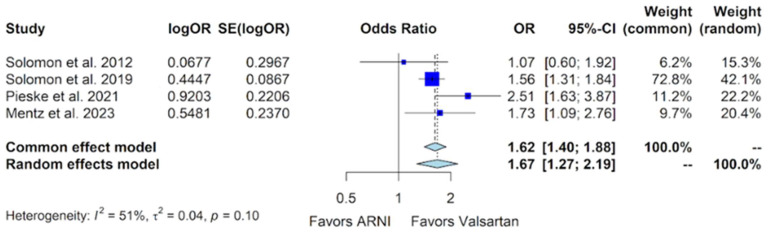
Forest plot showing the adverse event of hypotension with sacubitril/valsartan compared to valsartan in patients with HFmrEF and HFpEF. HFmrEF—heart failure with mildly reduced ejection fraction, HFpEF—heart failure with preserved ejection fraction, and ARNI—angiotensin receptor-neprilysin inhibitor. OR—odds ratio, SE—standard error, CI—confidence interval. In the forest plot, dark blue squares represent the point estimates, and the size of the square is a function of the weight given to each study in the meta-analysis. Horizontal solid black lines represent 95% CI. The bottom light blue diamonds represent the summary estimates, with the width of the diamond illustrating the 95% CI [10,11,12,13].

An analysis of common adverse events associated with sacubitril/valsartan, including hypotension, hyperkalemia, and worsening renal function, was conducted. The side effect of hypotension was investigated across all four studies. In the PARAMOUNT trial, 28 hypotension events were reported in the 149 patients (18.8%) within the sacubitril/valsartan group, compared to 27 events in the 152 patients (17.8%) in the valsartan group [10]. The PARAGON-HF trial reported 380 hypotension events among 2407 patients (15.8%) in the sacubitril/valsartan group compared to 257 out of 2389 (10.8%) in the valsartan group, with a significant *p*-value of <0.001 [11]. The PARALLAX trial reported 74 hypotension events out of 586 (12.6%) in the sacubitril/valsartan group compared to 32 out of 588 (5.4%) in the valsartan group [12]. The PARAGLIDE-HF trial reported 56 hypotension events out of 233 patients (24.0%) in the sacubitril/valsartan group compared to 36 out of 233 (15.5%) in the valsartan group [13]. In a pooled analysis of these data, evidence of heterogeneity was observed, prompting the adoption of a random-effects model. The analysis revealed a statistically significant risk of hypotension with sacubitril/valsartan compared to valsartan, with an OR of 1.67 (95% CI of 1.27 to 2.19, *p*-value of <0.0001) (Figure 8).

**Figure 9:**
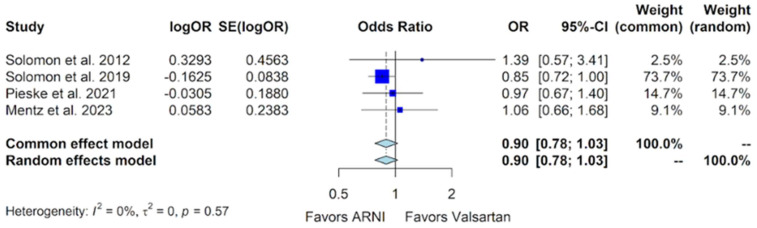
Forest plot showing the adverse event of hyperkalemia with sacubitril/valsartan compared to valsartan in patients with HFmrEF and HFpEF. HFmrEF—heart failure with mildly reduced ejection fraction, HFpEF—heart failure with preserved ejection fraction, and ARNI—angiotensin receptor-neprilysin inhibitor. OR—odds ratio, SE—standard error, CI—confidence interval. In the forest plot, dark blue squares represent the point estimates, and the size of the square is a function of the weight given to each study in the meta-analysis. Horizontal solid black lines represent 95% CI. The bottom light blue diamonds represent the summary estimates, with the width of the diamond illustrating the 95% CI [10,11,12,13].

The adverse outcome of hyperkalemia was reported in all four trials. In the PARAMOUNT trial, 12 hyperkalemia events were documented out of 149 patients in the sacubitril/valsartan group, compared to 9 out of 152 in the valsartan group, with a *p*-value of 0.50 [10]. The PARAGON-HF trial recorded 316 hyperkalemia events out of 2386 patients in the sacubitril/valsartan group and 361 out of 2367 patients in the valsartan group, with a *p*-value of 0.048 [11]. The PARALLAX trial reported 62 hyperkalemia events in the sacubitril/valsartan group of 586 patients, compared to 64 out of 588 patients in the valsartan group [12]. The PARAGLIDE-HF trial reported 45 hyperkalemia events out of 233 patients in the sacubitril/valsartan group, compared to 43 out of 233 in the valsartan group [13]. Upon a pooled analysis, there was no significant risk of hyperkalemia in the sacubitril/valsartan group compared to a placebo, with an OR of 0.90 (95% CI of 0.78 to 1.03, *p*-value of 0.124) (Figure 9).

**Figure 10:**
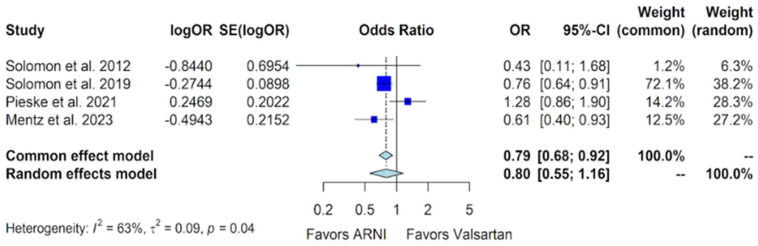
Forest plot showing the adverse event of worsening renal function with sacubitril/valsartan compared to valsartan in patients with HFmrEF and HFpEF. HFmrEF—heart failure with mildly reduced ejection fraction, HFpEF—heart failure with preserved ejection fraction, and ARNI—angiotensin receptor-neprilysin inhibitor. OR—odds ratio, SE—standard error, CI—confidence interval. In the forest plot, dark blue squares represent the point estimates, and the size of the square is a function of the weight given to each study in the meta-analysis. Horizontal solid black lines represent 95% CI. The bottom light blue diamonds represent the summary estimates, with the width of the diamond illustrating the 95% CI [10,11,12,13].

The adverse outcome of worsening renal function was documented in all four trials. In the PARAMOUNT trial, 3 such events occurred out of 149 patients in the sacubitril/valsartan group, compared to 7 events out of 152 patients in the valsartan group, with a *p*-value of 0.34 [10]. The PARAGON-HF trial reported 261 events of worsening renal function out of 2407 patients in the sacubitril/valsartan group, compared to 328 out of 2389 patients in the valsartan group, with a significant *p*-value of 0.002 [11]. The PARALLAX trial reported 61 events in the 586 patients included in the sacubitril/valsartan group, compared to 49 events out of 588 in the valsartan group [12]. The PARAGLIDE-HF trial reported 50 events out of 233 patients in the sacubitril/valsartan group compared to 72 out of 233 patients in the valsartan group [13]. In pooled analyses of these data, evidence of heterogeneity was observed, prompting the use of a random-effects model. The analysis indicated no statistically significant difference in the incidence of worsening renal function between the sacubitril/valsartan and valsartan groups, with an OR of 0.80 (95% CI of 0.55 to 1.16, *p*-value of 0.241) (Figure 10).

It is worth noting that, as indicated above, we observed heterogeneity in the safety outcomes related to hypotension and worsening renal function. Interestingly, the results from the PARALLAX trial (Pieske et al., 2021) differed somewhat from those of the other three trials, although the reasons for this difference are unclear. However, when the analyses were restricted to the three other trials, no evidence of heterogeneity was found (*p*-values for heterogeneity for the outcomes of hypotension = 0.41, worsening renal function = 0.48). In these analyses, sacubitril/valsartan again demonstrated a significantly higher risk of hypotension (OR 1.54, 95% CI 1.32 to 1.79, *p*-value < 0.0001), while showing a significantly lower risk of worsening renal function (OR 0.73, 95% CI 0.62 to 0.86, *p*-value = 0.0001). Forest plots illustrating these findings are available in the supplementary material.

## 4. Discussion

Several studies have investigated the efficacy of sacubitril/valsartan in patients with HFrEF, with the PARADIGM-HF trial being a pivotal contribution [6]. This trial demonstrated the superiority of sacubitril/valsartan over enalapril in reducing the risks of death and hospitalization for HF in patients with HFrEF. Specifically, sacubitril/valsartan significantly reduced the composite endpoint of cardiovascular death or HF hospitalization by 20% relative to enalapril in this patient population [6]. 

The PIONEER-HF trial further supported the utility of sacubitril/valsartan in HFrEF by revealing that, among patients hospitalized for acute decompensated HF, sacubitril/valsartan led to a greater reduction in the N-terminal pro-B-type natriuretic peptide (NT-proBNP) concentration compared to enalapril therapy [7]. The TRANSITION trial, an open-label study on patients with HFrEF hospitalized with worsening HF, demonstrated that the safety outcomes were similar for patients starting sacubitril/valsartan either before or after discharge, suggesting that early initiation may simplify management [8].

These pivotal studies have influenced clinical guidelines, with the 2022 AHA/ACC/HFSA guidelines recommending ARNI in patients with HfrEF and NYHA class II and III symptoms to reduce morbidity and mortality, receiving a Class I recommendation with Level A evidence [9]. In addition to the mentioned evidence, several meta-analyses in patients with HfrEF have consistently showed a lower risk of all-cause mortality and cardiovascular death in patients taking sacubitril/valsartan compared to ACEIs and ARBs [16,17,18,19]. However, these analyses also indicated an increased risk of hypotension with sacubitril/valsartan.

Along similar lines, a few RCTs were conducted in patients with HFmrEF and HFpEF to assess the impact of sacubitril/valsartan, although the results have not been as promising as those observed in trials focusing on HFrEF patients. The PARAMOUNT trial, led by Solomon et al., a phase 2 trial, demonstrated an improvement in NT-ProBNP with sacubitril/valsartan compared to valsartan in patients with HFpEF [10]. Conversely, the PARAGON-HF trial by Solomon et al. reported that sacubitril/valsartan did not significantly lower the rate of total hospitalizations for HF and cardiovascular death among patients with HF and an ejection fraction of 45% or higher [11]. The PARALLAX trial by Pieske et al. revealed that, among patients with HFmrEF and HFpEF, sacubitril/valsartan treatment, compared with ACEIs and ARBs or a placebo, resulted in a significantly greater decrease in plasma NT-ProBNP levels at 12 weeks. However, it did not significantly improve the 6 min walk distance at 24 weeks [12]. The PARAGLIDE-HF trial by Mentz et al. concluded that, among patients with HFmrEF and HFpEF, sacubitril/valsartan led to a greater reduction in plasma NT-proBNP levels and was associated with clinical benefits compared with valsartan alone [13].

A few meta-analyses have explored the effects of sacubitril/valsartan in patients with HFmrEF and/or HFpEF. Qin et al. conducted a meta-analysis of HFmrEF patients, comparing sacubitril/valsartan with ACEIs or ARBs. The findings suggested that sacubitril/valsartan may be an effective and safe strategy to improve left ventricular function, enhance quality of life, and reduce readmission rates [20]. Notably, the meta-analysis included 16 studies, with 15 originating from China, specifically on HFmrEF patients. Nielsen et al. performed a study investigating both HFrEF and HFpEF cohorts. For the HFpEF group, the study concluded that sacubitril/valsartan did not exhibit evidence of a significant difference compared with valsartan [18]. Another meta-analysis by Zhang et al. included patients with both HFrEF and HFpEF. The study revealed that, when compared with ACEIs or ARBs, sacubitril/valsartan significantly decreased the risk of death from all causes or cardiovascular causes and reduced hospitalization for HF in patients with HFrEF [19]. However, it failed to demonstrate an improvement in all-cause mortality and cardiovascular mortality in HFpEF cohorts. While these studies offer valuable insights into HFmrEF and HFpEF, they do have limitations, such as the inclusion of studies focusing on HFrEF, retrospective study designs, a limited number of studies on HFmrEF and HFpEF, and constraints on the range of outcomes that could be estimated.

Our study distinguishes itself from the aforementioned meta-analyses by exclusively and comprehensively examining RCTs specifically centered on the outcomes of sacubitril/valsartan compared to valsartan in patients with HFmrEF and HFpEF. This approach offers a more precise and detailed understanding of the benefits and risks associated with sacubitril/valsartan in this specific patient population. 

Our study revealed a small but significant improvement in the KCCQ CSS for sacubitril/valsartan compared to valsartan in patients with HFmrEF and HFpEF, with a mean difference of 1.13 (95% CI of 0.15 to 2.11) (Figure 3). Notably, in one of the studies (PARAGON-HF) contributing to the pooled analysis, we observed a drop (rather than an improvement) in the KCCQ CSS. [11] However, it was noteworthy that this decline was less pronounced in the sacubitril/valsartan group compared to the valsartan group. Despite this observation, our findings demonstrate a better KCCQ CSS in the sacubitril/valsartan group. 

Our study indicates favorable outcomes in the sacubitril/valsartan group with an improvement in the NYHA class, exhibiting an OR of 1.32 (95% CI of 1.10 to 1.59) compared to valsartan (Figure 4). Additionally, for the composite outcome of hospitalizations for HF and cardiovascular death in patients with HFmrEF and HFpEF, sacubitril/valsartan demonstrated superiority over valsartan, with a relative risk (RR) of 0.86 (95% CI of 0.75 to 0.99) (Figure 5). However, our study, in alignment with findings from Nielsen et al. and Zhang et al., indicates that there is no additional benefit with sacubitril/valsartan compared to valsartan concerning cardiovascular death and all-cause mortality [18,19]. Notably, our study incorporates data from Mentz et al., which were not included in the other meta-analyses [13].

This meta-analysis also explored the common side effects associated with both sacubitril/valsartan and valsartan in patients treated for HFmrEF and HFpEF. Our findings indicate a significant risk of hypotension with sacubitril/valsartan compared to valsartan, as evidenced by an OR of 1.67 (95% CI of 1.27 to 2.19) (Figure 8). To address the issue of heterogeneity that was noted in this analysis of hypotension (*p*-value for heterogeneity = 0.10), a forest plot was generated by excluding data from the PARALLAX trial. The subsequent analysis (*p*-value for heterogeneity = 0.41) reaffirmed a notable risk of hypotension (OR 1.54, 95% CI 1.32 to 1.79, *p*-value < 0.0001) [supplementary material, Figure S1] with sacubitril/valsartan, consistent with the original findings. The heightened risk of hypotension demonstrated in these analyses could be attributed to sacubitril/valsartan’s additional natriuretic effect compared to valsartan. Practical strategies for mitigating this risk may include dose adjustments of other diuretics or antihypertensive medications. 

The side effects of hyperkalemia and worsening renal function did not exhibit statistical significance in the sacubitril/valsartan vs. valsartan comparison, with ORs of 0.90 (95% CI of 0.78 to 1.03) and 0.80 (95% CI of 0.55 to 1.16), respectively (Figure 9 and Figure 10). However, after excluding the data from the PARALLAX trial to mitigate heterogeneity, the forest plot for worsening renal function revealed a reduced risk associated with sacubitril/valsartan (OR 0.73, 95% CI 0.62 to 0.86, *p*-value = 0.0001) [Supplementary Material, Figure S2].

Our study is subject to a few limitations. First, only four studies were eligible for inclusion due to strict inclusion criteria. Second, not all studies included in our meta-analysis provided data for every outcome assessed. For instance, outcomes such as changes in KCCQ CSS, improvements in NYHA class, and the composite outcomes of hospitalization for HF and cardiovascular death, as well as individual outcomes of cardiovascular death, were each sourced from two of the four studies. Third, our analysis identified heterogeneity in the safety outcomes of hypotension and worsening renal function. We employed a random-effects model for these outcomes to address the heterogeneity. In addition, we generated forest plots for these outcomes, removing the PARALLAX trial to mitigate heterogeneity. Fourth, for the individual outcomes of cardiovascular death and all-cause mortality, the analyses were based on simple counts, without factoring in the follow-up times. 

Despite these limitations, our study offers a more detailed analysis of various efficacy and safety outcomes related to HF treatment with sacubitril/valsartan compared with valsartan in patients with HFmrEF and HFpEF, providing a comprehensive and nuanced perspective of this medication. This study delves into distinct HF outcomes, including KCCQ CSS and improvements in NYHA class, which were not explored in previous meta-analyses. Furthermore, it provides insights into other HF outcomes, including a composite of hospitalization for HF and cardiovascular death and the individual outcomes of cardiovascular death and all-cause mortality, alongside common side effects. We believe this study enriches our understanding of the role of ARNI in these patient groups. Future large-scale, long-term RCTs focusing specifically on ARNI in the HFmrEF and HFpEF populations, either separately or combined, and exploring various laboratory and clinical HF outcomes and side effects would enhance clarity regarding its efficacy and utility in these patient cohorts.

## 5. Conclusions

This research shows that, in individuals with HFmrEF or HFpEF, in comparison with valsartan, sacubitril/valsartan can result in significant improvements in the HF outcomes of the KCCQ CSS, the NYHA class, and the composite outcomes of hospitalization for HF and cardiovascular death. However, no discernible advantage was observed in the specific outcomes of cardiovascular death and all-cause mortality. While there was a higher risk of hypotension in this patient population with sacubitril/valsartan compared to valsartan, this increased risk was not evident in the outcomes of hyperkalemia and worsening renal function. 

## Figures and Tables

**Table 1:** Table showing the characteristics of the studies included in the meta-analysis.

Sr No	Trial Name	Type of Study	Study Duration	Ejection Fraction	Study Population	Study Location	Sacubitril/Valsartan (N)	Valsartan (N)
1	Solomon et al., 2012 (PARAMOUNT) [10]	Phase 2, randomized, parallel-group, double-blind trial	36 weeks	≥45%	Age ≥ 40 years, documented history of HF with associated signs and symptoms, NT-proBNP > 400 pg/mL, on diuretic therapy, SBP < 140 mm Hg, or ≤160 mm Hg if on three or more BP medications, eGFR of ≥30 mL/min/1.73 m^2^	65 centers in 13 countries	149	152
2	Solomon et al., 2019 (PARAGON-HF) [11]	Randomized, double-blind, active comparator trial	4 years	≥45%	Age ≥ 50 years, with signs and symptoms of heart failure, NYHA class II to IV, EF ≥ 45% within the previous 6 months, elevated NT-proBNP, evidence of structural heart disease, and diuretic therapy	848 centers in 43 countries	2407	2389
3	Pieske et al., 2021 (PARALLAX) [12]	Randomized, double-blind, parallel-group clinical trial	24 weeks	≥40%	Age ≥ 45 years, with symptomatic heart failure, on diuretics, NYHA class II to IV, NT-proBNP ≥ 200 pg/mL (sinus rhythm) and ≥600 pg/mL (atrial fibrillation or flutter), with structural heart disease, KCCQ CSS < 75	396 centers in 32 countries	586	588
4	Mentz et al., 2023 (PARGLIDE-HF) [13]	Double-blind, randomized controlled trial	20 months	≥40%	Age ≥ 18 years with a diagnosis of HF, elevated BNP or NT-proBNP, with current hospitalization for WHF or within 30 days of a WHF	100 centers in the US and Canada	233	233

Table legend: HF—heart failure, NT-proBNP—N-terminal pro-B-type natriuretic peptide, SBP—systolic blood pressure, BP—Blood pressure, eGFR—estimated glomerular filtration rate, NYHA—New York Heart Association, KCCQ CSS—Kansas City cardiomyopathy questionnaire clinical summary score, WHF—worsening heart failure, and N—number of patients.

## Data Availability

Data sharing is not applicable. All the data are included in this original article, and no other new data were created or analyzed in this study.

## Supplementary Materials

The following supporting information can be downloaded at: https://www.mdpi.com/article/10.3390/jcm13061572/s1. Figure S1: Forest plot showing the adverse event of hypotension with sacubitril/valsartan compared to valsartan in patients with HFmrEF and HFpEF after removing the PARALLAX trial (Pieske et al. 2021 [12]). HFmrEF—heart failure with mildly reduced ejection fraction, HFpEF—heart failure with preserved ejection fraction, ARNI—angiotensin receptor-neprilysin inhibitor. Figure S2: Forest plot showing the adverse event of renal failure with sacubitril/valsartan compared to valsartan in patients with HFmrEF and HFpEF after removing the PARALLAX trial (Pieske et al. 2021 [12]). HFmrEF—heart failure with mildly reduced ejection fraction, HFpEF—heart failure with preserved ejection fraction, ARNI – angiotensin receptor-neprilysin inhibitor.
