# Supplementary material for: The Efficacy and Safety of Sacubitril/Valsartan Compared to Valsartan in Patients with Heart Failure and Mildly Reduced and Preserved Ejection Fractions: A Systematic Review and Meta-Analysis of Randomized Controlled Trials
Source: J Clin Med. 2024 Mar 9;13(6):1572. doi: 10.3390/jcm13061572 (PMC10971386; doi:10.3390/jcm13061572)
Supplement: Supplementary file 1 [file jcm-13-01572-s001.zip › jcm-2846760-supplementary.pdf]

Supplementary material

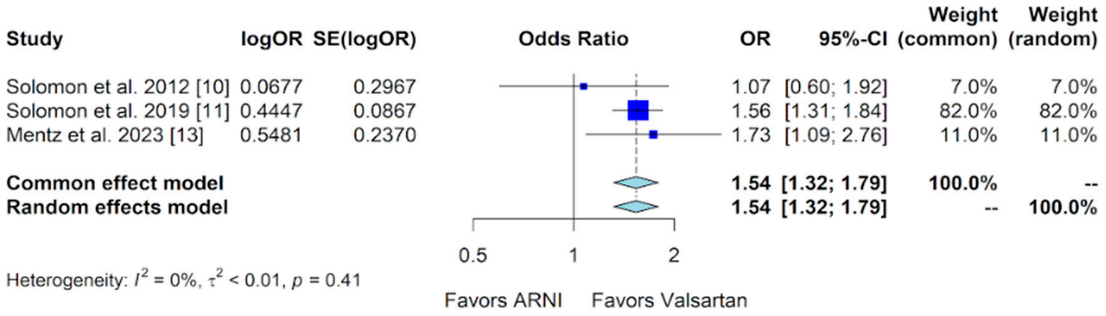

Figure S1: Forest plot showing the adverse event of hypotension with sacubitril/valsartan compared to valsartan in patients with HFmrEF and HFpEF after removing the PARALLAX trial (Pieske et al. 2021 [12]). HFmrEF – heart failure with mildly reduced ejection fraction, HFpEF – heart failure with preserved ejection fraction, ARNI – angiotensin receptor-neprilysin inhibitor, OR – odds ratio, SE – standard error, CI – confidence interval. In the forest plot, dark blue squares represent the point estimates, and the size of the square is a function of the weight given to each study in the meta-analysis. Horizontal solid black lines represent 95% CI. The bottom light blue diamonds represent the summary estimates, with the width of the diamond illustrating the 95% CI.

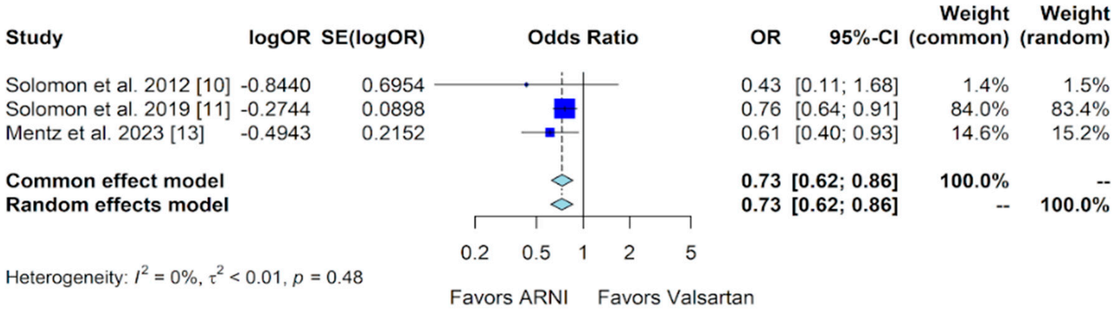

Figure S2: Forest plot showing the adverse event of renal failure with sacubitril/valsartan compared to valsartan in patients with HFmrEF and HFpEF after removing the PARALLAX trial (Pieske et al. 2021 [12]). HFmrEF – heart failure with mildly reduced ejection fraction, HFpEF – heart failure with preserved ejection fraction, ARNI – angiotensin receptor-neprilysin inhibitor, OR – odds ratio, SE – standard error, CI – confidence interval. In the forest plot, dark blue squares represent the point estimates, and the size of the square is a function of the weight given to each study in the meta-analysis. Horizontal solid black lines represent 95% CI. The bottom light blue diamonds represent the summary estimates, with the width of the diamond illustrating the 95% CI.
